# Deficits in Duration Estimation in Individuals Aged 10–20 Years Old with Idiopathic Mild Intellectual Disability: The Role of Inhibition, Shifting, and Processing Speed

**DOI:** 10.3390/ejihpe15080156

**Published:** 2025-08-11

**Authors:** Elsa Gourlat, Anne-Claire Rattat, Cédric T. Albinet

**Affiliations:** Laboratoire Sciences de la Cognition, Technologie, Ergonomie (SCoTE), Université de Toulouse, INU Champollion, 81012 Albi, Cedex 9, France; anne-claire.rattat@univ-jfc.fr (A.-C.R.); cedric.albinet@univ-jfc.fr (C.T.A.)

**Keywords:** time perception, duration estimation, inhibition, processing speed, shifting, mild intellectual disability

## Abstract

Time perception, especially duration estimation, plays a crucial role in the organization of behavior across development. Despite its importance, the cognitive mechanisms underlying impaired duration estimation remain insufficiently explored. Recently, the role of cognitive functions, such as executive functions, has been demonstrated in duration estimation. In the present study, the duration estimation, inhibition, shifting, and processing speed performances of participants with idiopathic mild intellectual disability (MID) without associated disorders (N = 79), aged between 10 and 20 years, were compared with those of typical participants (N = 81). The results show that the individuals with MID had difficulties in all cognitive functions (with the exception of one shifting task). Moreover, our results highlight—for the first time—the role of inhibition abilities and processing speed not only in the increase in duration estimation abilities with age, but also in the deficits observed in MID. To conclude, deficits in duration estimation in MID are due to an impairment of other cognitive functions.

## 1. Introduction

Duration estimation is a complex cognitive process that involves the subjective evaluation of a range of time (Block & Zakay, 2006). Accurate duration estimation is essential for daily functioning. For example, it enables individuals to reproduce, anticipate, and compare the duration of everyday actions. Inaccurate temporal estimates can have negative consequences, such as delays in planned activities; difficulties in meeting deadlines; and managing time in various contexts, including work, education, and social interactions (Barkley et al., 2001).

Recent studies have highlighted duration estimation difficulties (Gourlat et al., 2023, 2024; Rattat & Collié, 2020), among other cognitive difficulties (i.e., conceptual area), in people with idiopathic mild intellectual disability (for a review, see Gourlat et al., 2024). Mild intellectual disability is a neurodevelopmental disorder characterized by difficulties in both intellectual and adaptative functioning (with an intelligence quotient (IQ) of 50 to 70 on a standardized test) that emerges during the developmental period (INSERM, 2016). Individuals with mild intellectual disability thus have a limited understanding of risk in social situations associated with immature social judgment (i.e., social area), an occupation that requires few or fewer cognitive skills (i.e., practical area), and a highly pragmatic problem-solving technique compared to typically developing individuals (i.e., conceptual area) (APA, 2013). The prevalence of mild intellectual disability in France (1 to 2%; APA, 2013; INSERM, 2016) and in the world (1% in 2009; Maulik et al., 2011) is high, and 80% of all mild intellectual disability is idiopathic, indicating that its etiology is unknown (INSERM, 2016). However, the clinical profile of cognitive disabilities associated with idiopathic mild intellectual disability is largely imprecise; therefore, more work is needed to better understand this disorder and define specific care for this population. A previous study (Gourlat et al., 2023) supported the hypothesis that the difficulties in duration estimation in individuals with mild intellectual disability may be largely due to their lower working memory updating abilities. Furthermore, recent work has shown that adaptive behavior is predicted by executive functions (Gravråkmo et al., 2023). The present study focused on the link between duration estimation skills and other cognitive components, namely inhibition, shifting, and processing speed. The objective was to examine to what extent these cognitive functions are implicated in the development process of duration estimation and to what extent they can explain the difficulties in duration estimation in mild intellectual disability.

In the present study, we investigated the links between the developmental trajectories of duration estimation and inhibition, shifting, and processing speed in typically developing children and adolescents and those with mild intellectual disability aged 10 to 20 years. Indeed, the literature shows that executive functions develop until early adulthood (e.g., Tervo-Clemmens et al., 2023; Younger et al., 2023), processing speed until adolescence (e.g., R. V. Kail et al., 2016), and duration estimation until a child is old, at which point they are comparable to those of adults (e.g., Droit-Volet & Zélanti, 2013; Li et al., 2023).

Various tasks can be used to assess the ability to estimate durations, like the reproduction task that has already been used in numerous studies with children (e.g., Droit-Volet & Zélanti, 2013; Gautier & Droit-Volet, 2002; Hallez, 2020; Hallez et al., 2019; Hallez & Droit-Volet, 2017; Rattat & Chevalier, 2020). In this task, the participant must reproduce a target duration that has been previously presented to him/her. We intended to examine, from a developmental perspective, whether the difficulties in estimating durations in children and adolescents with mild intellectual disability, compared to typically developing children and adolescents, are related to not only lower updating abilities but also lower inhibition, shifting, and/or processing speed abilities.

Duration estimation processes can be theorized using the internal clock model, which asserts the existence of an internal mechanism for measuring time based on the perception of stimuli and environmental events (Gibbon et al., 1984). According to this model, duration estimation depends on the number of pulses generated continuously by a pacemaker and transferred into an accumulator through a switch, which can be opened or closed, although the transfer of pulses can only be performed when it is closed (for a review, see Wearden, 2003). The second level of processing, the memory level, corresponds to the storage of duration in memory. The duration representation is initially stored short-term in the working memory before eventually being stored in the longer-term reference memory if it is relevant to a task. Thus, when reproducing a previously presented duration, the closed switch enables pulses to be accumulated and counted until the total number of pulses reaches that previously stored in the memory. In addition, in the third and final level, the decisional level, an individual must compare one or more durations stored in their reference memory with the duration in their working memory to make a temporal judgment. For example, in a temporal reproduction task, an individual must reproduce as accurately as possible the reference duration of a previously presented stimulus. When this reference duration is presented, the individual accumulates a certain number of impulses (internal clock level). This information is then transferred to the working memory (memory level) and then compared to the reference duration (decisional level). Within the framework of the internal clock model, being able to accurately track the accumulation of pulses from the pacemaker likely requires executive resources, i.e., cognitive processes that optimize performance and identify the most appropriate behaviors to achieve a specific goal through the selection and simultaneous activation of different processes (Gilbert & Burgess, 2008). Numerous studies have highlighted significant correlations between performance on executive tasks and performance on duration estimation tasks (e.g., Baudouin et al., 2006; Brown, 2006; Droit-Volet & Zélanti, 2013; Ogden et al., 2011, 2014, 2019; Rattat, 2010; Rattat & Chevalier, 2020; Zélanti & Droit-Volet, 2011). In particular, Ogden et al. (2014) suggested that the duration reproduction task is more executively demanding than other temporal tasks, such as discrimination tasks, and reported a significant correlation between duration reproductions and performance on both working memory updating and shifting tasks, but not on inhibition tasks. More recently, Rattat and Chevalier (2020) showed that inhibitory control is required to reproduce durations accurately and that the improved ability to accurately reproduce durations with age may be due to the development of executive abilities, particularly inhibitory abilities.

Regarding the involvement of inhibition resources in duration estimation, the literature actually offers somewhat contradictory results, with some studies highlighting a link between inhibition resources and duration estimation (e.g., Brown, 2006; Brown et al., 2013; Rattat & Chevalier, 2020), while others do not (e.g., Fortin et al., 2010; Ogden et al., 2011, 2014). Inhibition is a cognitive process that involves suppressing automatic or dominant responses or thoughts (for a review, see Diamond, 2013). The contradictory results on inhibition may be partly due to the task used, as inhibition effects on duration estimations are more accentuated in more complex tasks (Brown et al., 2013). As inhibition plays a crucial role in regulating attentional resources, we can assume that individuals with inhibition difficulties may struggle to filter out irrelevant stimuli, which increases the cognitive load and reduces the attentional resources available for estimating durations (e.g., Block et al., 2010; Droit-Volet & Zélanti, 2013). Diminished attentional resources can lead to more interference, disrupting the functioning of the internal clock’s switch, which controls the allocation of attention to temporal information (Block & Zakay, 1997).

Consequently, individuals may have difficulties in accurately perceiving and processing durations. Inhibition difficulties can also impair updating of the working memory processes involved in the storage and manipulation of temporal information. Indeed, duration estimation requires updating resources as a part of online maintenance, time tagging, and replacing information in the working memory (e.g., Brown & Frieh, 2000; Ogden et al., 2011). In a reproduction task, the participant must monitor and maintain two durations in their memory (the target duration and the reproduced duration). Disruptions in inhibition may hinder these processes, leading to inaccurate duration estimation and memory retrieval (Barkley et al., 2001).

Similarly, high shifting performances have been associated with high accuracy in a temporal reproduction task (e.g., Brown et al., 2013; Wearden et al., 2010). Shifting corresponds to the ability to alternate between multiple tasks, rules, or thoughts and, thus, to adapt to different situations (for a review, see Diamond, 2013). In such a task, shifting resources are required to alternate between the reproduced duration and the memory representation of the target duration (Ogden et al., 2014) in order to compare them and determine when the reproduced duration should be stopped. Thus, according to the internal clock model, deficits in shifting can also impair duration estimation.

In individuals with mild intellectual disability, studies have revealed that they have difficulties in both their inhibition and shifting processes (e.g., Danielsson et al., 2012; Spaniol & Danielsson, 2022; Van der Molen et al., 2007; Zagaria et al., 2021). More precisely, specific inhibitory control difficulties associated with mild intellectual disability have been noted in young children aged 4 to 7 years (Grada & Simoni, 2018), older children aged 10 to 14 years (Gligorović & Buha Ðurović, 2014), and adolescents aged 15 to 23 years (Bexkens et al., 2014; Zagaria et al., 2021). These individuals do not mobilize their inhibitory resources in the same way as typically developing individuals (i.e., preserved emotional inhibition and deficient behavioral inhibition) (Treillet et al., 2014), and this inhibition deficit correlates with externalized behavior disorders in individuals with mild intellectual disability (Schuiringa et al., 2017). However, these studies had methodological limitations, such as the lack of a control group (Gligorović & Buha Ðurović, 2014; Grada & Simoni, 2018; Schuiringa et al., 2017) and the inclusion of participants with different levels of intellectual disability (moderate intellectual disability, Treillet et al., 2014; borderline intellectual disability, Bexkens et al., 2014; Schuiringa et al., 2017), which could have affected the interpretation of the results. Regarding shifting skills, while some works have shown no link between shifting performance and IQ (e.g., Friedman et al., 2006), others have reported a deficit in shifting abilities (Delis et al., 2001) in individuals with an intellectual disability (e.g., Erostarbe-Pérez et al., 2022) and mild intellectual disability (e.g., Gligorović & Buha, 2013). Danielsson et al. (2012) showed that the shifting abilities of 11-to-15-year-old children with mild intellectual disability on a verbal fluency task were lower than those of typically developing individuals matched on chronological age. Contrary to the studies that have used shifting tasks that also involved various cognitive processes, such as inhibition or short-term memory, a recent study (Kitamura et al., 2022) that used a shifting task with a minimal involvement of cognitive processes other than shifting (the Truck Loading Task developed by Fagot & Gauvain, 1997) showed that shifting abilities in individuals with mild intellectual disability do not differ from those of typically developing individuals matched on both mental and chronological age, despite a higher variability in the performance of the individuals with mild intellectual disability. Thus, considering the few studies and the heterogeneous results on shifting, it is difficult to clearly conclude whether this process is preserved or altered in mild intellectual disability. Further studies are needed to clarify this issue.

To sum up, compared to typically developing individuals, individuals with mild intellectual disability show a lower performance on inhibition tasks. The results are more heterogeneous for shifting skills, as they appear to be more task-dependent. From a developmental point of view and despite some controversy, the development of different executive components (inhibition and shifting) appears to mature relatively independently in typically developing individuals until adolescence (for a recent review, see Theodoraki et al., 2020). No study has yet looked at their development from childhood to adulthood in individuals with mild intellectual disability.

The speed with which information is processed is a cognitive skill frequently associated with executive functions (R. Kail & Salthouse, 1994; Miyake et al., 2000). The information processing speed refers to the efficiency with which individuals can perceive, interpret, and respond to incoming information (Salthouse, 1996). It is considered a low-level process that underlies other more complex cognitive functions, such as executive functions (Lee et al., 2013; Miyake et al., 2000). Droit-Volet and Zélanti (2013) also demonstrated that the information processing speed is the best predictor of individual variations in time sensitivity (i.e., temporal variability). More precisely, their results showed that a faster information-processing speed was associated with participants’ higher sensitivity to time (i.e., less variable time performance). Insofar as the speed of information processing is slower in individuals with a low IQ (Loranger et al., 2000) and in individuals with intellectual disability (R. Kail, 1992), we can therefore expect to observe a lower performance on a reproduction task among individuals with mild intellectual disability than among typically developing individuals.

The current research adopted a developmental perspective to assess the duration estimation, inhibition, shifting, and information processing speed in a cohort of typically developing individuals and those with idiopathic mild intellectual disability aged 10–20. Their duration estimation abilities were assessed using a reproduction task. Their inhibition and shifting abilities were each analyzed using three tasks to mitigate problems of task impurity and to investigate individual performance with respect to the cognitive construct. As Rabbitt (1997) and Miyake et al. (2000) argued, measuring an executive function directly is problematic because it operates within other cognitive processes. Thus, to address these challenges and ensure a more reliable measure, composite scores for probing inhibition and shifting derived from the three experimental tasks had to be computed. However, as explained below in the Materials and Methods section and discussed in the Discussion section, we were not able to use these composite scores because these two theoretical constructs revealed low internal consistency. As a result, we were obliged to examine each task individually. An important point is that all the tasks used in the present study were given to the participants of all age ranges, facilitating the examination of the developmental trajectory of duration estimation, inhibition, shifting, and information processing speed abilities not only in typically developing individuals but also in those with idiopathic mild intellectual disability.

Consequently, this approach made it possible to investigate the interaction between the cognitive abilities of interest as a function of age and mild intellectual disability status. Drawing on the existing literature, we expected to demonstrate difficulties in the cognitive abilities (i.e., inhibition, shifting, information processing speed) of individuals with idiopathic mild intellectual disability and a delayed developmental trajectory of these capacities compared to chronologically age-matched typically developing children and adolescents. Additionally, we hypothesized a significant link between cognitive performance and performance on the reproduction task, with the individuals with idiopathic mild intellectual disability showing a decreased difficulty in reproducing the duration after accounting for their cognitive difficulties.

## 2. Materials and Methods

### 2.1. Participants

The present study included the same participants as in a previous study on the implications of working memory updating for duration estimation capacities in typically developing children and adolescents and those with and idiopathic mild intellectual disability aged 10 to 20 years (Gourlat et al., 2023). The final sample comprised 160 participants, including 81 typically developing individuals and 79 individuals with idiopathic mild intellectual disability without associated disorders. The principal characteristics of this final sample are presented in Table 1.

As described in Gourlat et al. (2023), all participants were recruited from five primary and secondary educational institutions, as well as universities, along with ten medico-social establishments located in the southwest of France, more specifically in Occitanie. Each participant’s respective institution directly confirmed the diagnosis of idiopathic mild intellectual disability. Rigorous screening procedures were implemented to ensure that the individuals with idiopathic mild intellectual disability were not diagnosed with any co-existing disorder(s), such as dyslexia, dysorthographia, Attention Deficit Hyperactivity Disorder (ADHD), or Autism Spectrum Disorder (ASD), nor did they manifest any other neurological or genetic pathologies. Additionally, the age of the typically developing participants had to correspond with their respective levels of education. Individuals with any sensory impairments related to vision, perception, or motor skills that would likely interfere with the performance on the experimental tasks were excluded.

Prior to participation, informed consent was obtained from the participants and, in the case of minors, from their parents. The consent of the directors of the schools and medico-social establishments was also obtained.

### 2.2. Materials and Tasks

As mentioned previously, the present study is part of a larger study on duration estimation abilities and executive functions in typically developing individuals and those with mild intellectual disability. The present study encompassed eight tasks. The first session included a temporal reproduction task (already described in a previous manuscript, Gourlat et al., 2023) and an information processing speed task (i.e., choice reaction time task). The second session comprised three inhibition tasks (i.e., Go no-Go task, Real Animal Size Task, Multisource Interference Task), while the third session incorporated three shifting tasks (i.e., Wisconsin Card Sorting Test, Alternative Run Task Shifting, and Cued Task Shifting). The task sequences within the sessions and session order were counterbalanced across the participants. The only exception was that session 1 was performed first. All the tasks were adapted from pre-existing validated tasks, tailored not only to accommodate the clinical characteristics of mild intellectual disability but also to suit the participants’ age ranges, ensuring the uniform management of tasks. Stimuli were presented, and the responses were recorded on a laptop via a homemade program developed in Python programming language (version 3.9) using the PsychoPy library (version 2021.1.0;). The participants’ responses were recorded using two-millisecond-accurate response boxes: a Cedrus RB-740 response box was used in sessions 1 and 3, and a custom-built (4 × 4) 16-key response box was utilized in session 2 (further details are provided below).

### 2.3. Evaluation of Duration Estimation Abilities

**Duration Reproduction Task**. The full description of this task can be found in Gourlat et al. (2023). Briefly, in this task, the participants had to reproduce the reference duration of a stimulus (from 400 to 800 ms) as accurately as possible. The dependent variables (DVs) for this task were the accuracy score, corresponding to the mean difference between the temporal reproduction and stimulus duration divided by the stimulus duration (from −1 to 1; a score close to 0 is associated with better performance), and the variability score (standard deviation/mean of accuracy score).

### 2.4. Evaluation of Inhibition Abilities

**Go No-Go Task (GNG)**. Inspired by the work of Chevalier et al. (2014), the participants in this task had to press the response box as quickly as possible after the presentation of a target stimulus (a yellow or red fish), but inhibit the response (not press the response box) when a non-target stimulus (a blue or purple fish) appeared on the screen. The participants used two buttons on the response box to perform the task: a green button to place themselves in the fishing boat and a blue button to catch the fish. First, they had to hold down the green button to place themselves in the boat. Subsequently, they were presented with the next trial (the fish appears). If they wished to catch the fist, they could release the green button (exit the boat) and press the blue button (catch the fish). Finally, they returned to the boat (green button) for the next trial. When a distractor appeared, the participants had to hold down the green button (stay in the boat) and not press the blue button. This task consisted of a demonstration phase, a training phase, and a test phase comprising 4 blocks of 40 trials. Each block contained 10 Go trials and 30 no-Go trials. The position of the key on the box (right or left) and the fish not to be caught (4 combinations) were counterbalanced. The DV for this task was the inhibition sensitivity score (d’). Higher d’ was associated with better performance. The reliability score for this task was 0.98 (split-half method).

**Multisource Interference Task (MIT)**. Inspired by Bush and Shin’s study (Bush & Shin, 2006), in this task, participants were given a keyboard with three keys in a row, representing the numbers 1, 2, and 3. A series of three numbers appeared every 2 s in the center of the screen. One digit was always different from the other two. Participants had to identify which number was different and press the corresponding key. Two types of blocks were performed: control blocks and interference blocks. In control blocks, the target number always corresponded to its position on the response button (for example, the number “1” would appear in the first position). In interference blocks, the target never corresponded to its position on the response button, and distractors were themselves potential targets (e.g., 233, the correct answer is “2”). This task consisted of a demonstration phase, a training phase, and a test phase comprising 18 blocks of 24 trials (9 control blocks and 9 interference blocks). The DV for this task was the interference cost of reaction time (RT difference between interference and control blocks in ms). A lower interference was linked to better performance. The reliability score for this task was 0.99 (split-half method).

**Real Animal Size Task (RAST)**. Adapted from Rattat and Chevalier’s study (Rattat & Chevalier, 2020), in this task, two drawings of different animals of varied sizes (one large and one small) were presented side by side in each trial. The participants were asked to press a key on the side that showed the animal that was larger in real life. In this test, two situations were tested: congruent trials, where the animal that was taller in real life was also visually taller on the screen, and incongruent trials, where the larger animal was depicted as visually smaller, creating interference between the real-life size and visual size. In these trials, the participants had to inhibit the visually salient size on the screen to respond to the size of the animal in real life. This task consisted of a demonstration phase, a training phase, and a test phase comprising 48 trials (24 congruent trials and 24 incongruent trials) presented in a random order, with an inter-trial interval of 1500 ms. The DV was the interference cost (RT incongruent–RT congruent in ms). A lower interference cost was associated with better performance. The reliability score for this task was 0.98 (split-half method).

### 2.5. Evaluation of Shifting Abilities

**Wisconsin Card Sorting Test Task (WCST)**. Based on Cianchetti et al.’s study (Cianchetti et al., 2007), the participants had to sort 48 cards according to a rule (shape, color, or number), placing the card on the screen onto one of the 4 piles shown at the top of the screen. The sorting rule changed after 6 correct categorizations. The shifting performance was essentially assessed by the number of sorting errors and, in particular, perseveration errors (continuing to apply an obsolete rule). This task consisted only of a test phase comprising 48 trials. If the participants managed to successfully identify all 3 criteria (shape, color, and number) before the end of the task, it stopped. If not, the task stopped after 48 trials. The DV for this task was the number of perseverative errors. A higher number of perseverative errors was associated with lower performance.

**Alternative Run Shifting Task (ARTS)**. Adapted from Karayanidis et al.’s study (Karayanidis et al., 2013), in this task, successive stimuli appeared at 4 different screen locations (top right, top left, bottom left, and bottom right). A different rule had to be applied depending on where they appeared. The stimuli were either a leaf or a flower shown on the top, along with either a bird or a fish shown on the bottom. The stimuli appeared clockwise, one after the other. The shifting performance was essentially assessed by the percentage of correct responses and the calculation of the temporal cost of shifting. The task consisted of a univalent phase for the vegetation (leaf or flower on the top—2 blocks of 16 trials), a univalent phase for the animals (bird or fish on the bottom—2 blocks of 16 trials), a bivalent phase for the vegetation (the participant faced all the stimuli in all locations but responded only to the vegetation stimuli—2 blocks of 16 trials), a bivalent phase for the animals (the participant faced all the stimuli in all locations but responded only to the animal stimuli—2 blocks of 16 trials), and a mixed phase comprising both the bivalent vegetation and animal stimuli (the participant faced all the stimuli in all locations and responded to both the vegetation and animal stimuli—4 blocks of 16 trials). The DV for this task was the shifting cost (RT from switched trials–RT from repeated trials in ms). A lower shifting cost was associated with higher performance. The reliability score for this task was 0.95 (split-half method).

**Cued Task Shifting (CTS)**. Based on Chevalier et al.’s study (Chevalier et al., 2018), the participants were presented with four toys to sort according to the shape (car or teddy bear) or color (blue or pink). The participants had to arrange the toys according to the specific rule (color or shape) presented on the screen. The task consisted of two non-switch phases (the color phase and the shape phase) and a mixed phase composed of both color and shape trials (switched trials). In each phase, feedback appeared after the answer was given (green or red smiley). There was no time limit, although the participants were asked to perform it as quickly and correctly as possible. Specifically, they had to put the object in the correct box by pressing one of the two illuminated buttons on the boxes. This task consisted of a color phase (2 blocks of 16 trials), a shape phase (2 blocks of 16 trials), and a mixed phase (8 blocks of 16 trials). The order of the two first phases (color or shape) was counterbalanced. The DV for this task was the shifting cost (switch RT–non-switched RT in ms). A lower shifting cost was linked to higher performance. The reliability score for this task was 0.98 (split-half method).

### 2.6. Evaluation of Information Processing Speed

**Choice Reaction Time Task (CRT)**. Based on Albinet et al.’s (2012) work, the participants were asked to respond as quickly and accurately as possible to one of two stimuli presented: if the arrow displayed was pointing to the right (>), the participant pressed the right key with their right hand, and if the arrow was pointing to the left (<), they had to press the left key with their left hand. The arrows were displayed on the computer screen until a response was given. Before each trial, a fixation point (+) was briefly presented. This task included a demonstration phase, a training phase, and a test phase comprising 2 blocks of 44 trials. The DV for this task was the RT (ms). A shorter RT was associated with higher performance. The reliability score for this task was 0.99 (split-half method).

### 2.7. Computation of a Composite Score Reflecting Inhibition and Shifting

Cronbach’s alphas were computed from the z-scores (using the means and standard deviations for the whole group) for the inhibition tasks and from the z-scores for the shifting tasks to assess how well each three tasks of a component measured a single underlying construct. Unfortunately, the Cronbach’s alphas were too low to compute a relevant composite score (inhibition: α = 0.507; shifting: α = 0.094). Accordingly, we used the raw scores for each task instead of the composite score.

### 2.8. Procedure

The participants completed their individual testing sessions in a quiet, isolated environment over three separate sessions, with a minimum interval of 30 min between sessions to ensure adequate rest. Positioned in front of a computer screen and a response box, the participants were seated on a chair during testing. Before beginning, the experimenter provided detailed instructions regarding the test procedures, ensuring the participants’ comprehension. The anonymized participant data were recorded on the testing computer prior to initiation. Throughout the sessions, adherence to epidemic-related barrier measures was rigorously maintained, including disinfection of the equipment between participants and mandatory mask usage. If the participants exhibited any signs of discomfort or unease, testing could be halted, although such instances did not occur. On average, each session lasted between 30 and 45 min, including scheduled breaks.

### 2.9. Statistical Analyses

The main aims of the present study were to verify that children and adolescents with mild intellectual disability present with deficits in inhibition, shifting, and processing speed performance compared to typically developing chronological age-matched children and adolescents and to examine the extent to which these cognitive deficits may account for their deficits in duration estimation abilities. To this end, we first conducted multiple analyses of variance (MANOVAs) followed by univariate analyses of variance (ANOVAs) on each DV of the seven cognitive tasks (three tasks examining inhibition, three tasks examining shifting, and one task examining information processing speed), with the group (mild intellectual disability vs. typically developing) and age (10–12, 13–16, and 17–20 years) as the between-subjects factors. For the MIT and CRT tasks, we controlled for the effect of gender (ANCOVAs with 1 = male; 2 = female), as the main effect of gender was significant for these tasks. The post hoc analyses used Bonferroni corrections for multiple comparisons. Second, we conducted bivariate partial correlations (controlling for gender) between each cognitive measure and the two performance indices of the duration reproduction task. Finally, we ran multiple linear regression analyses involving the cognitive measures, which demonstrated a significant correlation with the measures of the temporal performance as predictors. The significance level was set at *p* ≤ 0.05. The partial estimated effect sizes (η^2^_p_) were reported for significant effects.

## 3. Results

### 3.1. Duration Estimation

#### 3.1.1. Temporal Reproduction Task

The results for this task were already presented in a previous manuscript (Gourlat et al., 2023). We summarize below the main results relevant to the present study and refer the interested reader to the original article for details.

- ***Accuracy Score***. The participants with mild intellectual disability demonstrated significantly less accuracy in the reproduction of durations. In particular, while all the participants overestimated the shortest durations (400 to 530 ms), this temporal overestimation was more marked in the participants with mild intellectual disability (0.31 ± 0.55) than in the typically developing ones (0.13 ± 0.28). Despite the accuracy score significantly differing as a function of age, none of the 2–2 comparisons showed any significant differences between the age groups (10–12 years old: 0.31 ± 0.55; 13–16 years old: 0.15 ± 0.43; and 17–20 years old: 0.20 ± 0.33).
- ***Variability Score***. Overall, the variability score was significantly lower for the 17–20-years-old group (0.36 ± 0.17) compared to the two younger groups, which did not differ from each other (10–12 years old: 0.42 ± 0.20; 13–16 years old: 0.43 ± 0.22). Moreover, the participants with mild intellectual disability consistently showed greater variability in their temporal reproductions (0.49 ± 0.23) compared to the typically developing participants (0.31 ± 0.12).

#### 3.1.2. Executive Functions

- ***Inhibition.*** The MANCOVAs (controlling for gender) conducted on the three inhibition tasks revealed significant main effects of group (Wilks’ lambda = 0.466; *F*(3,146) = 57.7, *p* < 0.0001) and age (Wilks’ lambda = 0.738; *F*(6,292) = 8, *p* = 0.03), as well as a significant age × group interaction effect (Wilks’ lambda = 0.91; *F*(6,292) = 2.35, *p* < 0.0001). The results of the univariate ANOVAs for each task are reported below.

**Figure 1:**
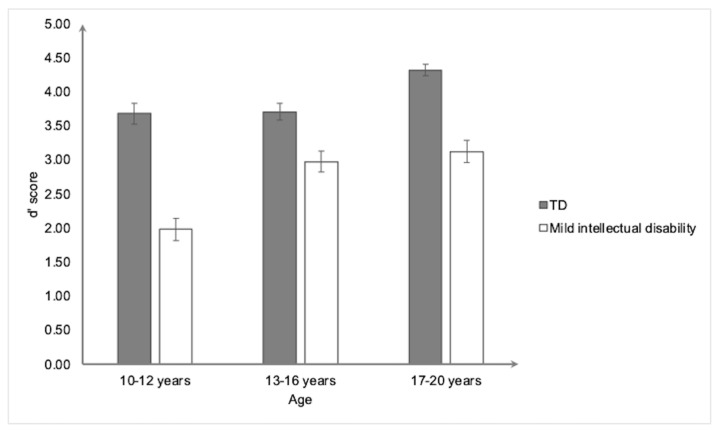
Mean inhibition sensitivity score (d’) on the Go No-Go task as a function of group and age. Bars represent standard errors.

For the GNG task (Figure 1), the ANOVA revealed a significant main effect of group, *F*(1,154) = 106.86, *p* < 0.001. The η^2^_p_ = 0.41, showing that the participants with mild intellectual disability (2.71 ± 0.96) had a lower d’ score than the typically developing participants (3.90 ± 0.70). The ANOVA also revealed a significant main effect of age, *F*(2,154) = 19.32, *p* < 0.001, and η^2^_p_ = 0.20. The post hoc analyses revealed that all the age groups differed from each other, with d’ increasing with age (10–12 years vs. 13–16 years, *p* < 0.05; 10–12 years vs. 17–20 years, *p* < 0.001; and 13–16 years vs. 17–20 years, *p* < 0.05). Finally, the group ×age interaction effect was also significant, *F*(2,154) = 5.62, *p* < 0.05, and η^2^_p_ = 0.068. In both the mild intellectual disability and typically developing groups, the main effect of age was significant (*F*(2,76) = 14.46, *p* < 0.001, η^2^_p_ = 0.28; and *F*(2,78) = 8.53, *p* < 0.001, η^2^_p_ = 0.18, respectively).

In the mild intellectual disability group, the 10–12-year-olds had a lower inhibition sensitivity score than both the 13–16- (*p* < 0.001) and the 17–20-year-olds (*p* < 0.001). In the typically developing group, the 17–20-year-olds had a higher inhibition sensitivity score than both the 13–16- (*p* < 0.05) and the 10–12-year-olds (*p* < 0.05). Furthermore, the main effect of the group was significant in each of the three age groups, showing that the participants with mild intellectual disability consistently obtained a lower d’ score compared to the typically developing participants (10–12 years old: *F*(1,50) = 57.48, *p* < 0.001, η^2^_p_ = 0.54; 13–16 years old: *F*(1,52) = 13.57, *p* < 0.001, η^2^_p_ = 0.21; and 17–20 years old: *F*(1,52) = 42.13, *p* < 0.001).

**Figure 2:**
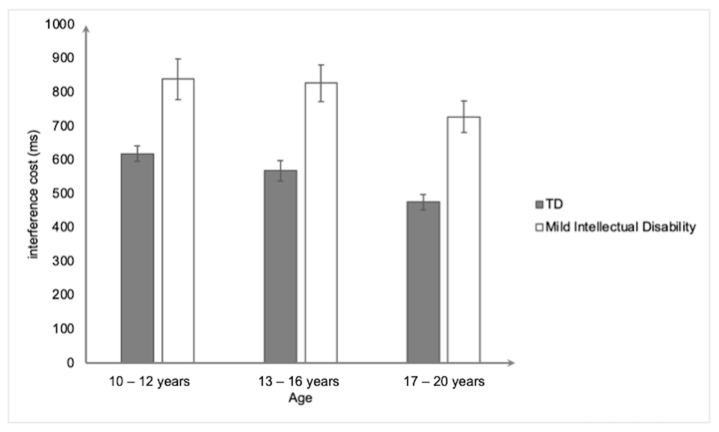
Mean interference cost (in ms) on the Multisource Interference Task as a function of group and age. Bars represent standard errors.

For the MIT (Figure 2), the ANCOVA (controlling for gender) revealed only a significant main effect of group, *F*(1,153) = 43.68. *p* < 0.001, and η^2^_p_ = 0.22, showing that the participants with mild intellectual disability had a higher interference score (797 ms ± 277) than the typically developing participants (554 ms ± 143). The main effect of age and the group × age interaction effect were insignificant (both *ps* > 0.05).

**Figure 3:**
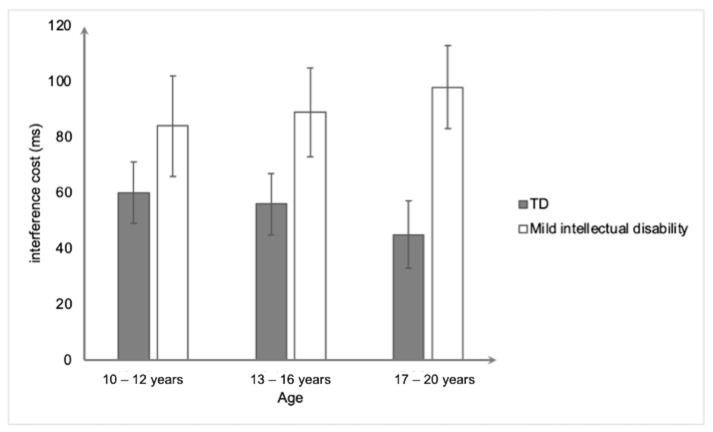
Mean interference cost (in ms) on the Real Animal Size Task as a function of group and age. Bars represent standard errors.

For the RAST (Figure 3), the ANOVA also revealed only a significant main effect of group, *F*(1,154) = 10.49, *p* < 0.001, and η^2^_p_ = 0.064. The participants with mild intellectual disability obtained a higher interference score (90 ms ± 81) than the typically developing participants (54 ms ± 58). The main effect of age *(p* > 0.05) and the interaction effect between group and age were significant (*p* > 0.05).

- ***Shifting.*** The MANOVAs conducted on the three shifting tasks revealed a significant main effect of group (Wilks’ lambda = 0.793; *F*(3,152) = 13.2, *p* < 0.0001). The results of the univariate ANOVAs for each task are reported below.

**Figure 4:**
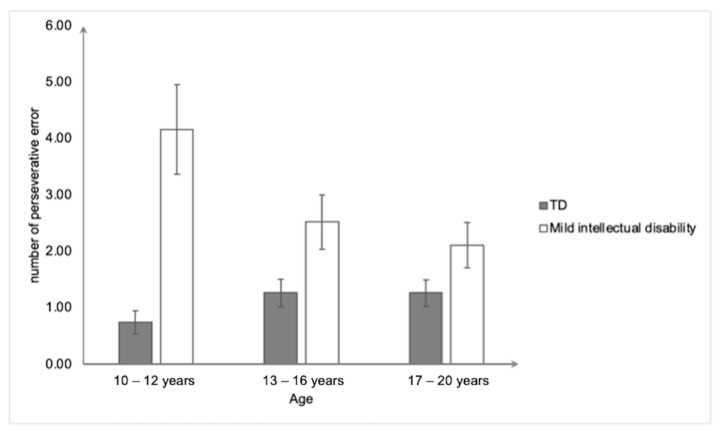
Mean number of perseverative errors on the Wisconsin Card Sorting Task as a function of group and age. Bars represent standard errors.

For the WCST (Figure 4), the main effect of the group was significant, *F*(1,154) = 28.10, *p* < 0.001, and η^2^_p_ = 0.15, revealing that the participants with mild intellectual disability made more perseverative errors (2.90 ± 0.34) than the typically developing participants (1.09 ± 1.16). The group × age interaction effect was also significant, *F*(2,154) = 5.18, *p* < 0.01, and η^2^_p_ = 0.063. The main effect of age for the participants with mild intellectual disability was significant, *F*(2,76) = 3.58, *p* < 0.05, and η^2^_p_ = 0.086. More precisely, the 10–12-years-old group made significantly more perseverative errors than the 17–20-years-old group (*p* < 0.05). By contrast, the main effect of age among the typically developing participants was insignificant, *F*(2,78) = 1.82, *p* > 0.05. Furthermore, the participants with mild intellectual disability made more perseverative errors than the typically developing participants in the 10–12-years-old group, *F*(1,50) = 19.14, *p* < 0.001, η^2^_p_ = 0.028; and in the 13–16-years-old group, *F*(1,52) = 5.62, *p* < 0.05, η^2^_p_ = 0.098; but not in the 17–20-years-old group, *F*(1,52) = 3.39, *p* > 0.05.

**Figure 5:**
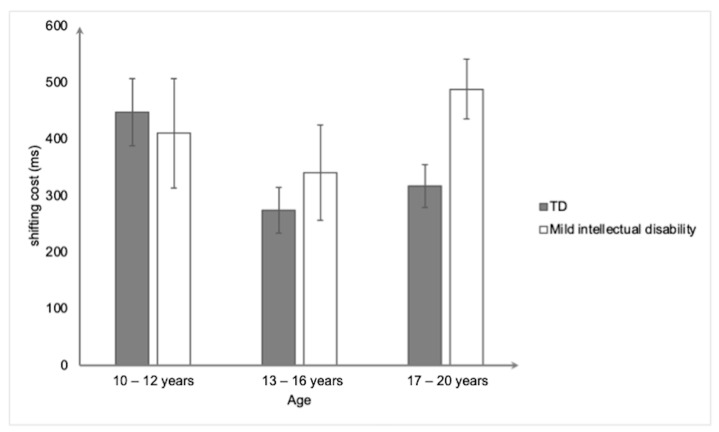
Mean shifting cost (in ms) on the Alternating Running Task Switch as a function of group and Age. bars represent standard errors.

For the ARTS (Figure 5), the ANOVA did not reveal any significant main or interaction effects (all *ps* > 0.1).

**Figure 6:**
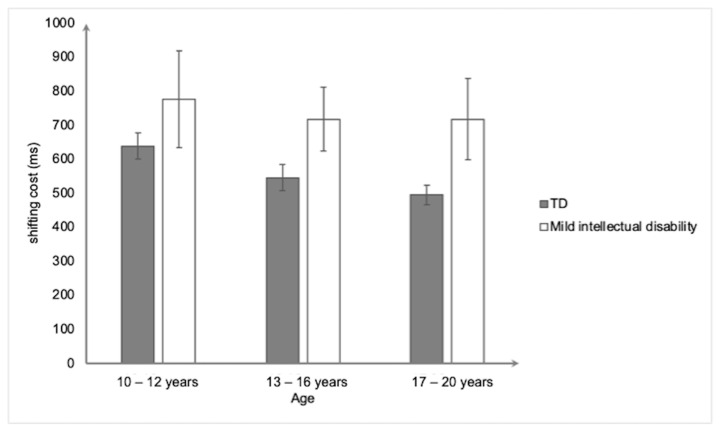
Mean shifting cost (in ms) on the Cued Task Switching as a function of group and age. Bars represent standard errors.

Regarding the CTS (Figure 6), the ANOVA revealed a significant main effect of group, *F*(1,154) = 6.23. *p* < 0.05, and η^2^_p_ = 0.04, indicating that the participants with mild intellectual disability had a higher shifting cost (736 ms ± 604) than the typically developing participants (560 ms ± 195). No other effect was significant (all *Fs* < 1).

**Figure 7:**
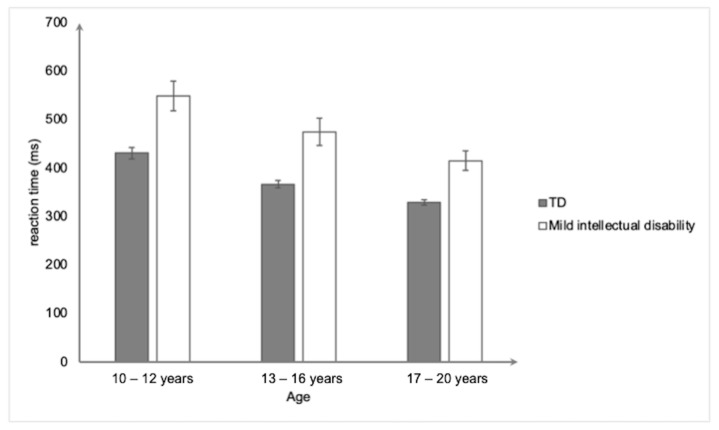
Mean reaction time (in ms) on the Choice Reaction Time task as a function of group and age. Bars represent standard errors.

- ***Information processing speed*.** The results of the ANCOVA (controlling for gender) revealed a significant main effect of group, *F*(1,153) = 48.63. *p* < 0.001, and η^2^_p_ = 0.24, showing that the participants with mild intellectual disability had a longer reaction time (478 ms ± 145) than the typically developing participants (375 ms ± 61) (Figure 7). The ANCOVA also revealed a significant main effect of age, *F*(2,153) = 21.26. *p* < 0.001, and η^2^_p_ = 0.22. The post hoc analyses showed that the 17–20-year-old group had a significantly shorter reaction time compared to both the 13–16- (*p* < 0.05) and the 10–12 (*p* < 0.001)-year-old groups. These two latter groups also significantly differed from each other (*p* < 0.05). Finally, the group × age interaction effect was not significant (*F* < 1).

#### 3.1.3. Relationship Between Duration Estimation and Cognitive Performance

The results of the bivariate partial correlations (controlling for gender) showed that the performance on the GNG task was significantly and negatively correlated with both the accuracy score (r = −0.37, *p* < 0.001) and the variability score (r = −0.50, *p* < 0.001) on the time duration reproduction task (Table 2). The reaction time on the CRT task was positively correlated with both the accuracy score (r = 0.56, *p* < 0.001) and the variability score (r = 0.39, *p* < 0.001). The performance on the MIT was positively correlated only with the variability score (r = 0.27, *p* < 0.001). Finally, the number of perseverative errors on the WCST was also positively correlated with the variability score (r = 0.26, *p* < 0.001). These findings indicate that the participants with better inhibition performance on the GNG task and faster information processing speeds reproduced the durations more accurately and with less variability. The participants with better inhibition performance on the MIT and with better cognitive shifting performance on the WCST also reproduced the durations with less variability.

Subsequently, we performed a series of multiple linear regression analyses to examine the extent to which the performance on these cognitive tasks accounted for the variance observed in the accuracy and variability scores on the time duration reproduction task as a function of group and age. First, we ran a multiple linear regression analysis with the accuracy score on the reproduction task as the dependent variable, the group and age as the fixed factors, the d’ score on the GNG task and the reaction time on the CRT task as the predictors, and gender as a covariate. The model was significant (*F*(5,154) = 15.1, *p* < 0.001) and accounted for 33% of the total variance (R^2^ = 0.33). The follow-up analysis showed that the variables group and age were insignificant (both *ps* > 0.05), and that only the reaction time on the CRT task (β = 2.03, *p* < 0.001) but not the d’ on the GNG task (*p* > 0.1) was a significant predictor. In other words, the variations in information processing speed explained most of the variance in the accuracy score on the duration reproduction task, due to the presence or absence of mild intellectual disability and increased age.

Second, we ran a multiple linear regression analysis with the variability score on the duration reproduction task as the dependent variable and the group and age as the fixed factors; the d’ score on the GNG task, the reaction time on the CRT task, the interference cost on the MIT, and the number of perseverative errors on the WCST as the predictors; and gender as a covariate. The model was significant (*F*(7,152) = 13.1, *p* < 0.001) and accounted for 38% of the total variance (R^2^ = 0.38). The follow-up analysis indicated that the group and age were no longer significant (both *ps* > 0.05), and that only the d’ on the GNG task (β = −0.06, *p* < 0.001) was a significant predictor. The three other predictors were not significant (all *ps* > 0.05). In other words, the variations in inhibition performance explained most of the variance in the variability score on the duration reproduction task due to the presence or absence of mild intellectual disability and increased age.

## 4. Discussion

This study compared children and adolescents with idiopathic mild intellectual disability with typically developing individuals regarding the development of inhibition, shifting, and information processing speed abilities, and explored the association between these variables and the participants’ duration reproduction abilities. Our findings highlighted that the individuals with mild intellectual disability had lower inhibition, shifting, and information processing speed performances than the typically developing individuals on all tasks except the one task evaluating shifting (ARTS). Furthermore, the performance of the participants with mild intellectual disability improved with age across three tasks, inhibition (GNG task), shifting (WCST), and information processing speed (CRT task), while among the typically developing participants it improved on only two of them (GNG and CRT). Moreover, the multiple linear regression analyses revealed that the information processing speed measured by the CRT task explained that most of the differences in the accuracy performance on the duration reproduction task were due to mild intellectual disability and age, and that the inhibition abilities measured by the GNG task explained that most of the differences in the variability performance on the duration reproduction task were due to mild intellectual disability and age. To better understand these results, we will focus, respectively, on the development of the link between duration reproduction and inhibition, shifting, and information processing speed in mild intellectual disability before discussing some important theoretical and methodological limitations of our study.

Regardless of age, the children and adolescents with mild intellectual disability demonstrated lower inhibition performances than their chronological age-matched peers on all three tasks. This result is consistent with previous studies showing an inhibition deficit in individuals with mild intellectual disability aged between 4 and 23 years (e.g., Gligorović & Buha Ðurović, 2014; Grada & Simoni, 2018; Zagaria et al., 2021). Importantly, even at an age of 17 to 20 years, the individuals with mild intellectual disability in our study showed a lower inhibition performance on the three inhibition tasks than the typically developing individuals aged 10 to 12 years. The inhibition performance also improved with age in both the individuals with mild intellectual disability and the typically developing individuals, but only for the GNG task, although the developmental trajectory differed between the two groups. More precisely, in the individuals with mild intellectual disability, their inhibition performance increased from 10–12 years to 13–16 years and then stagnated, while in the typically developing individuals, it stagnated from 10–12 years to 13–16 years and then increased from 13–16 years to 17–20 years. Overall, the individuals with mild intellectual disability improved earlier than the typically developing individuals, but their level of performance remained low, and even at ages 17–20, they did not catch up with typically developing individuals aged 10–12. The inhibition skills of adults with mild intellectual disability over 20 years old should be examined to determine whether they are fully developed or still evolving. The developmental curve observed in our groups of typically developing individuals is consistent with that in the literature, which suggests a sharp increase in inhibition at preschool age, then a slower increase through adolescence, and complete maturation around age 17 (Best & Miller, 2010; Chevalier, 2010; Fourneret & des Portes, 2017). However, this age effect was found only for the GNG task and not for the MIT or RAST, which might suggest that our three tasks require different inhibition resources. We will return to this point in detail in a later section.

Our results also highlighted that inhibition, as measured by the GNG task, was a significant predictor of duration reproduction performance variability. This means that the inhibition performance evaluated by this task explained most of the deficit in variability performance during the reproduction task due to mild intellectual disability and age. Although a previous study has already supported a link between inhibition and duration reproduction performance in typically developing children and adolescents (Rattat & Chevalier, 2020), the present study is the first to demonstrate this association in individuals with mild intellectual disability. How can we explain this link between the variability in duration reproductions and inhibition abilities? In the duration reproduction task, the participants had to accumulate temporal information and then monitor and maintain two durations (the target duration and the reproduced duration) in their memory. Therefore, in the context of the internal clock model, inhibition deficits may affect the switch functioning and retention of the duration in the working memory by ineffectively filtering out irrelevant stimuli. This inefficiency may include inconstant responses (high variability), i.e., difficulties in correctly reproducing the target duration in a time-consistent manner. Indeed, our results highlighted that the participants with mild intellectual disability exhibited greater variability in duration reproduction performance than the typically developing participants, which was explained by their lower inhibition performance as measured by the GNG task.

Our findings also showed a deficit in the shifting process in individuals with mild intellectual disability in two out of the three tasks used. More precisely, the participants with mild intellectual disability showed a lower shifting performance on the WCST and the CTS than the typically developing participants, while the two groups did not differ significantly on the ARTS. Globally, our results favor a deficit in the shifting abilities in this population. These results add to the current but limited and controversial literature on the effect of mild intellectual disability on shifting abilities, with some studies showing deficits (e.g., Danielsson et al., 2012; Gligorović & Buha, 2013), while one recent study showed somewhat preserved abilities (Kitamura et al., 2022). The shifting performance in mild intellectual disability might be task-dependent, as our results suggest, or may be linked to differences in other cognitive processes involved in the tasks we employed in the current study. Further studies are needed to clarify these points.

From a developmental perspective, shifting abilities improved with age, as measured by the WCST in the group with mild intellectual disability; specifically, the 17–20-year-old participants performed better than the 10–12-year-old participants. This is the first study to highlight this age-related improvement in shifting abilities in mild intellectual disability, although it pertains to only one out of the three tasks used. In the existing literature on typical development, some studies have demonstrated that shifting abilities appear around the age of 3, and progress slowly throughout adolescence (for a review, see Fourneret & des Portes, 2017). Therefore, we should have observed age-related differences, at least between the youngest and oldest participants in our typically developing sample, but this was not the case. Methodological reasons, discussed later, could partly explain these results.

Regarding the involvement of shifting abilities in duration reproduction ability, our results showed, in the first step, that the WCST performance was significantly correlated with the variability score for the duration reproduction task. This is consistent with previous reports (e.g., Ogden et al., 2014). However, when entered with the other measures of cognitive performance into a multiple linear regression analysis, the WCST performance was not a significant predictor of duration reproduction performance variability, regardless of age or mild intellectual disability. The only significant predictor in this analysis was the inhibition measure of the GNG task, as discussed above. This is important because, having measured several cognitive functions using various tasks, our study allowed us to delineate that, above shifting abilities, inhibition abilities are the most powerful and only significant predictor of differences in duration reproduction variability due to both age and mild intellectual disability. Such a conclusion could not be reached by previous studies, which assessed only one cognitive function at a time.

Finally, our findings revealed a slower information processing speed, as measured by the CRT, in individuals with mild intellectual disability than in typically developing individuals. This result is consistent with previous studies showing a slower information processing speed in individuals with low IQ (e.g., Loranger et al., 2000) and with an intellectual disability (e.g., R. Kail, 1992). The information processing speed performance also improved with age in both the typically developing participants and those with mild intellectual disability. Indeed, the 17-to-20-year-old adolescents had a faster information processing speed than those aged 13 to 16, who in turn showed a faster information processing speed than the younger group aged 10 to 12. These results are also largely consistent with the literature on typical development, which reports an increase in information processing speed between the ages of 9 and 12 and even up to the age of 15, when performance becomes similar to that of adults (e.g., Williams et al., 2016). However, in our study, the adolescents further improved their information processing speed after age 15, since the 17-to-20-year-old participants were the fastest. In line with previous studies (e.g., Droit-Volet & Zélanti, 2013; Fink & Neubauer, 2005; Perbal et al., 2002; Rammsayer & Brandler, 2007), we also highlighted a correlation between information processing speed, as measured by the CRT, and both the variability in and accuracy scores on the duration reproduction task.

Furthermore, when the information processing speed was controlled, the group and age effects previously observed on the accuracy score (but not on the variability score) of the reproduction task disappeared. This means that the information processing speed largely explained the accuracy deficit in the reproduction task due to mild intellectual disability and age. One potential explanation for this can be found in the neurophysiological literature. In 1961, Survillo showed that the speed of information processing increases with the speed of the alpha rhythm of neural oscillations. As this frequency increases with age, it might reflect the age-related increase in neural oscillatory activities that support time representation, suggesting that temporal precision is directly linked with information processing speed (Droit-Volet & Zélanti, 2013). Consequently, a faster information processing speed directly predicted better precision in duration reproduction and thus explained the better accuracy performance of older and typically developing individuals compared to individuals with mild intellectual disability.

To sum up, our results suggest that the lower inhibition skills of the participants with mild intellectual disability can explain the greater variability in their duration reproductions, while their slower information processing speed mainly explains the decreased accuracy of their temporal reproductions. However, these interpretations need to be discussed considering some of the limitations or unresolved questions of the present study. As outlined in the Introduction section, one of our main aims was to examine the influence of inhibition and shifting abilities at the level of cognitive construct and not at the level of experimental tasks to overcome the problem of task impurity (Miyake et al., 2000; Rabbitt, 1997). However, we were unable to find the expected common structure for both inhibition and shifting measures. Consequently, we had to conduct our analyses task by task, instead of using the factorial approach of executive functions. First, the GNG task appears to be the only executive task that mobilizes the motor dimension in our protocols, and the only one whose subjects’ performance is dependent on this motor aspect. The child presses the keys of a box to give their answer. They have to wait, so as not to press the key too soon, and at the right time. In his 2010 study, Droit-Volet showed that the overestimation of short durations in a temporal reproduction task was due to the motor components, which take longer in adults than in children (e.g., Droit-Volet, 2010), although the motor response is generated at the same time. This component would only be involved in the reproduction of short durations (2.5 s) and would not predict the reproduction performance for long durations (4.5 s). Thus, a lack of inhibition would induce motor difficulties in preventing oneself from responding quickly, leading to overestimations, but also to more variable responses depending on attentional fluctuations. This raises the question of the extent to which the temporal variability might be explained by the motor dimension of inhibition in the GNG task. This question of the unicity or diversity of executive functions is still debated in the literature. The most prominent models consider the existence of a common executive factor in the preschool period, which is categorized into three distinct factors (inhibition, cognitive flexibility, and updating working memory) from the age of 6 or later (Best & Miller, 2010; Diamond, 2013; Friedman et al., 2008; Lee et al., 2013; Miyake et al., 2000). More recently, some authors using network models found different effects across populations, depending on the executive task used to measure a target executive function, and they did not find a common factorial structure for each executive function (Holmén et al., 2024; Rosales et al., 2023). Shifting and inhibition do not sufficiently explain the variance in cognitive tasks. These findings support a revision of Miyake’s model and, more generally, question the assessment of executive functions using factorial models (for a review and meta-analysis, see Karr et al., 2018). Moreover, several studies have discussed the fact that inhibition and shifting are not unitary constructs by themselves (Ionescu et al., 2024; Nigg, 2017; Patil et al., 2025; Raud et al., 2020). In recent years, the literature has discussed the possibility of evaluating executive functions more ecologically through everyday activities that mobilizing several executive functions (Suchy et al., 2024a, 2024b; Wallisch et al., 2018).

Second, as we analyzed our results task by task, we found heterogeneous findings; the results concerning some tasks confirmed our expectations, while others did not. Although our tasks all had a very high reliability score, it is possible that they measured processes of different complexity, or processes that are somewhat different. There are contradictory results in the literature showing the involvement of cognitive resources in duration estimation (e.g., inhibition—Rattat & Chevalier, 2020; shifting—Danielsson et al., 2012) or not (e.g., inhibition—Ogden et al., 2011; shifting—Kitamura et al., 2022). According to the authors, these differences are due to the type of task or to the task complexity. This is consistent with recent studies that have questioned Miyake’s model and found the same structure (Holmén et al., 2024; Rosales et al., 2023). These heterogeneous findings are not always easy to interpret. For the inhibition tasks, we can assume that the GNG task requires more motor skills than the other two inhibition tasks and they are similar to the motor skills required in the duration reproduction task. As Droit-Volet (2010) demonstrated, this involvement of motor components may explain why the performance on this GNG task was related only to the performance on the duration reproduction task. Another possible explanation could be a ceiling effect for the MIT and RAST. Indeed, even if we did not report the proportion of correct responses for these two tasks, they were very high, reaching the maximum for many individuals, including the children and adolescents with mild intellectual disability. Our results showed very high success rates as many children, especially the typical ones, seemed to score 100%. In Rattat and Chevalier’s work (Rattat & Chevalier, 2020), the authors showed that between the ages of 7 and 10 and young adulthood, the percentage of correct answers on the RAST increased, although it is more significant between the ages of 7 and 10. Thus, it is possible that in our study between the ages of 10 and 21 the gap narrowed and we reached this ceiling effect.

For the shifting tasks used in our study, the different results between our three tasks could be due to the mobilization of cognitive processes other than shifting. For example, many studies have shown that the WCST requires other cognitive abilities, such as abstraction, working memory, inhibition, or planning (for a review, see Miles et al., 2021). Here, we can also postulate a ceiling effect for the CTS and the ARTS, as the proportion of correct responses to these tasks was also very high. Clearly, more work is still needed to disentangle these speculations.

## 5. Conclusions

Using a developmental experimental design and multiple regressions analyses, the present study mainly confirmed our hypotheses that children and adolescents with mild intellectual disability show deficits in duration estimation, inhibition, shifting, and information processing speed. Moreover, we showed that their lower inhibition and slower processing speed explains, to a large extent, these duration estimation deficits. More precisely, we highlighted that their lower inhibition skills mainly explain the greater variability in their duration reproductions. In contrast, their slower information processing speed mainly explains the lower accuracy of their temporal reproductions. These findings complement those of our previous study, which showed that working memory updating abilities also explain part of the temporal deficits in the same reproduction task (Gourlat et al., 2023). We can, therefore, now explore the hierarchy of the involvement of these different cognitive components in the duration estimation deficits of individuals with mild intellectual disability and their developmental trajectory. A limitation of the present study is that it relied only on a subset of the tasks used because the latent construct approach of executive functions could not be applied in this study. Further studies should also include adults older than 20 years to determine whether the cognitive difficulties observed in children and adolescent with mild intellectual disability persist to adulthood (suggesting a deficit) or are overcome later (suggesting a developmental delay).

## Figures and Tables

**Table 1:** Characteristics of the participant groups.

GROUP	AGE		
	10–12 Years Old	13–16 Years Old	17–20 Years Old
**Typically developing**			
**N**	27	27	27
**% boys (n)**	51.85 (14)	67.00 (18)	18.52 (5)
**mean age**	11.22	13.56	18.63
**sd**	0.64	0.89	1.11
**Mild Intellectual Disability**			
**N**	25	27	27
**% boys (n)**	76.00 (19)	59.26 (16)	55.56 (15)
**mean age**	10.76	14.56	18.11
**sd**	0.93	1.15	0.85

N = number; sd = standard deviation.

**Table 2:** Partial correlations (controlling for gender) between the performances on cognitive and duration reproduction tasks. * *p* < 0.01, *** *p* < 0.001.

Variables	Accuracy Score	Variability Score
GNG	r = −0.34 ***	r = −0.46 ***
MIT	r = 0.083	r = −0.21 *
RAST	r = −0.087	r = −0.14
WCST	r = 0.075	r = 0.26 ***
ARTS	r = −0.069	r = 0.06
CTS	r = −0.005	r = 0.14
CRT	r = 0.49 ***	r = 0.39 ***

GNG: Go no-Go; MIT: Multisource Interference Task; RAST: Real Size Animal Task; WCST: Wisconsin Card Sorting Test; ARTS: Alternating Run Task Switching; CTS: Cued Task Switching; CRT: Choice Reaction Time.

## Data Availability

The raw data supporting the conclusions of this article will be made available by the authors on request.
